# Academic achievements and brain volume development in children and adolescents

**DOI:** 10.1093/texcom/tgac048

**Published:** 2022-11-16

**Authors:** Teruo Hashimoto, Yutaka Matsuzaki, Susumu Yokota, Ryuta Kawashima

**Affiliations:** Division of Developmental Cognitive Neuroscience, Institute of Development, Aging and Cancer, Tohoku University, 4-1 Seiryomachi Aobaku, Sendai 980-8575, Japan; Division of Developmental Cognitive Neuroscience, Institute of Development, Aging and Cancer, Tohoku University, 4-1 Seiryomachi Aobaku, Sendai 980-8575, Japan; Kyushu University, Faculty of Arts and Science, 744 Motoaka, Nisiku, Fukuoka 819-0395, Japan; Division of Developmental Cognitive Neuroscience, Institute of Development, Aging and Cancer, Tohoku University, 4-1 Seiryomachi Aobaku, Sendai 980-8575, Japan

**Keywords:** calculation, magnetic resonance imaging, mathematics, voxel-based morphometry

## Abstract

Children are expected to acquire both basic and numeric skills. Achievement of higher levels of reading, writing, arithmetic, and vocabulary are favorable and desirable. The relationship between each literacy skill and neural development has been investigated; however, association between brain development and the 4 literacy skills has not been examined. This longitudinal, structural, neuroimaging study explored the contribution of higher academic achievement in reading, writing, arithmetic, and vocabulary to neural development. The brain volumes of children and adolescents aged 9–16 years were measured in the first test. Approximately 2.6 years later, the brain volumes and 4 academic achievement scores of 77 participants were measured in the second test. Changes in the gray matter volume in the left fusiform gyrus were associated with vocabulary scores, whereas those in the left striatum were associated with arithmetic scores. The reading and writing scores showed no statistically significant relationship with changes in brain volume. The current vocabulary score correlated with current gray matter volume, while brain volumes in the first test showed no association with any achievement scores. These results suggest that academic achievement may modulate brain plasticity in various ways.

## Introduction

Academic achievement encompasses basic literacy, vocabulary, and mathematical skills that are associated with children’s self-regulation ability (McClelland et al. 2007). Early mathematical and vocabulary skills aid in predicting subsequent school reading and mathematical achievements (Duncan et al. 2007). Furthermore, children’s vocabulary aids in predicting subsequent achievements in reading and mathematics (Morgan et al. 2015). Achievements in mathematics and reading at childhood are better predictors of socioeconomic status (SES) at middle age than intelligence and SES at childhood (Ritchie and Bates 2013). Also, SES can predict academic achievement (Sirin 2005). Higher academic achievements during childhood can benefit future health (Lê-Scherban et al. 2014). Therefore, understanding the mechanisms associated with higher academic achievement during childhood may provide useful insights for all age groups.

Neural mechanisms associated with higher intelligence, generally understood as aptitude (inherited cognitive ability) and learned knowledge measured with IQ tests, have been explored in children. Children with superior intelligence exhibit greater cortical thinning at adolescence than those with average intelligence (Shaw et al. 2006). At 10 years of age, children with higher IQ have a thinner cortex than children with lower IQ. A longitudinal study reported that verbal intelligence in children is associated with gray matter thinning in the left frontoparietal areas (Sowell et al. 2004). Cortical thinning during childhood, as detected using magnetic resonance imaging (MRI), is associated with greater myelination (Natu et al. 2019); whereas a decrease in gray matter in the elderly reflects neuronal loss (Terry et al. 1987). Gray matter volume (GMV) and cortical thickness in children aged > 8 years decrease until mid-adolescence (Gennatas et al. 2017; Mills et al. 2021). Reduced GMV is associated with higher academic achievement after mid-childhood.

Associations between academic achievement and brain development have also been previously reported. Cortical thinning in the left frontoparietal region is correlated with an improvement in vocabulary in children (Sowell et al. 2004). The cortical thickness in the left inferior parietal lobule is lower in children with higher vocabulary growth rates than in those with lower vocabulary growth rates (Asaridou et al. 2017). Left superior temporal thickness is associated with reading in children (Perdue et al. 2020). In a large sample size study, reading in children had less of an effect on cortical thickness; in contrast, reading in adolescents and young adults was associated with higher cortical thickness in the left inferior parietal lobule and fusiform gyrus than in other parts of the brain (Torre et al. 2020). Torre et al. (2020) also reported no associations between math calculation scores and broad brain areas, including the left inferior parietal lobule; this suggested dissociations in neuroanatomical correlation between reading and math. Writing performance can be associated with right inferior frontal volume in young children (Gimenez et al. 2014). Greater arithmetic performance in children is associated with a greater right fusiform gyrus volume (Polspoel et al. 2020). Higher math achievement is associated with a greater hippocampal volume in children (Wilkey et al. 2018). These cross-sectional studies indicate that academic achievement can enhance cortical development in various brain areas. Longitudinal studies of young children have indicated parietal involvements in mathematical abilities (Price et al. 2016; Kuhl et al. 2020; Skeide et al. 2020). Better reading skills are correlated with greater GMV decrease in the left inferior frontal, parietal, and sensorimotor areas in children (Houston et al. 2014; Linkersdorfer et al. 2015). However, longitudinal studies examining the relationship between academic achievement and brain development are limited.

This study aimed to examine the association between academic achievement and brain development in children and adolescents. Structural brain changes were measured using MRI during the first and second visits. Vocabulary, reading, writing, and arithmetic performances were measured using the achievement scales of a developmental test (Kaufman Assessment Battery for Children, Second Edition [KABC-II]) in the second visit. The relationships between regional GMV (rGMV) and achievement scores were examined using voxel-based morphometry (VBM) analyses. We hypothesized that children with higher achievement scores in each skill would show greater rGMV reductions in diverse brain regions than in those with lower achievement scores.

## Materials and methods

### Participants

First, 144 children and adolescents aged 6–18 years were recruited through a newspaper advertisement as control subjects for studies of developmental disorders (Hashimoto et al. 2020, 2021). For this study, 77 children and adolescents (46 boys and 31 girls) aged 9–16 years participated in the first test. As GMV has been reported to decrease approximately between 8 and 17 years of age (Gennatas et al. 2017; Mills et al. 2021), we selected 9–16 years of age for this study. Of these 77 children, 17 also participated in previous studies (Hashimoto et al. 2015b, 2016). Inclusion criteria were age 9–16 years in the first visit without intellectual disability in both the first and second visits (full-scale intelligence quotient, FSIQ < 70). Exclusion criteria were having any history of malignant tumors or head trauma involving loss of consciousness, epilepsy, impaired color vision, diagnosis of developmental disorders, routine visits to a hospital because of illness, congenital disorders, or routine use of medications (except for over-the-counter drugs such as cold or anti-allergy medications). After a mean of 2.6 years (32 months, range: 28–41 months), 77 children participated in the second visit. Data from 77 participants (46 boys and 31 girls) were obtained. All the children were native Japanese speakers with normal or corrected-to-normal vision and auditory functions. Seventy-four children were right-handed and 3 were left-handed. Handedness was determined using the Edinburgh Handedness Inventory (Oldfield 1971).

According to the Declaration of Helsinki, written informed consent was obtained from the parents of each participant before performing MRI scans. This study was approved by the Ethics Committee of the Tohoku University Medical School (2019-1-703).

### Intelligence and socioeconomic status measures

Brain images, IQ, and SES were collected during the first visit. Academic achievement scores, brain images, IQ, and SES were measured during the second visit. Trained examiners conducted IQ tests using the fourth edition of the Japanese version of the Wechsler Intelligence Scale for Children (WISC-IV) aged <  16 years and Wechsler Adult Intelligence Scale (WAIS-IV) for participants aged >  16 years.

SES was obtained from parents during both the first and second visits. The assessment of SES (range: 2–14) comprised inquiries related to annual family income (7 levels:1, <US $20,000 [the currency exchange rate was set at US $1 = 100 yen]; 2, US $20,000–$40,000; 3, US $40,000–$60,000; 4, US $60,000–$80,000; 5, US $80,000–$100,000; 6, US $100,000–$120,000; and 7, >US $120,000) and the educational qualifications of both parents (7 possibilities: 1, elementary school graduate or below; 2, junior high school graduate; 3, normal high school graduate; 4, graduate of a short-term school completed after high school (such as a junior college); 5, university graduate; 6, master’s degree; and 7, doctorate). For educational qualifications, the average of both parents was used.

### Academic achievement tests

We used the Japanese version of the KABC-II (Kaufman and Kaufman 2004). Vocabulary, reading, writing, and arithmetic scores were measured only during the second visit. During the first visit, the children and their parents were not informed about assessment of the academic achievement in the second visit. Standardized scores (mean = 100, SD = 15) for each age group were used in the analyses.

### Image acquisition

All images were collected using a 3 T Philips Intera Achieva scanner (Amsterdam, Netherlands). Three-dimensional, high-resolution T1-weighted images were collected using a magnetization-prepared rapid gradient-echo sequence. The parameters were as follows: 240 × 240 × 162 matrix, time repetition (TR) = 6.5 ms, time echo (TE) = 3 ms, time to inversion (TI) = 711 ms, field of view (FOV) = 24 cm, 162 slices, and 1.0-mm slice thickness (voxel size: 1.0 × 1.0 × 1.0 mm^3^). The scan duration was 8 min and 3 s. First, we checked the T1 image by visual on-site inspection; when obvious motion artifacts were found, the T1 image was scanned again immediately, and the re-scanned image was used for further analyses.

**Fig. 1:**
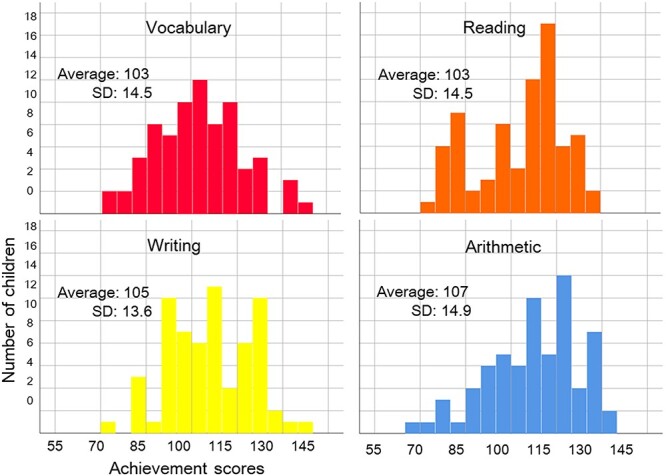
Distributions of achievement scores in the second visit.

### Preprocessing of MRI data

MRI data were preprocessed and analyzed for VBM using the statistical parametric mapping (SPM12) software (Wellcome Department of Cognitive Neurology, London, United Kingdom) with a computational anatomic toolbox (CAT12, http://www.neuro.uni-jena.de/hbm2016/GaserHBM2016.pdf). The CAT12 longitudinal data pipeline was used to calculate the rGMV. The CAT12 longitudinal data pipeline includes intra-subject realignment, bias correction, segmentation, and normalization, which are more sensitive to small structural changes (e.g. brain plasticity) than SPM12 longitudinal registration that can only detect neurodegenerative changes. Tissue probability maps and the ICBM space template of East Asian brains were applied. The T1-weighted images of each individual were segmented into 6 tissue segments using the default parameter settings of the segmentation algorithm. Diffeomorphic anatomical registration was performed using an exponential lie (DARTEL) algebraic registration process implemented in CAT12. The resulting images were spatially normalized to the Montreal Neurological Institute (MNI) space to obtain images with 1.5 × 1.5 × 1.5 mm^3^ voxels. Volume change correction (modulation) was performed by modulating each voxel with Jacobian determinants derived from spatial normalization, thus enabling the determination of regional differences in the absolute amount of brain tissue. The sample homogeneity of the preprocessed images was assessed. Furthermore, all images were smoothed by convolution with an isotropic Gaussian kernel of 8-mm full width at half maximum.

### Quality check of MRI data

For quality assurance of the MRI data, we used the image quality rating (IQR) that is the summary of segmentation results (resolution, noise, and bias) of CAT12 (http://www.neuro.uni-jena.de/cat/index.html#QA). The IQR is shown as a percentage; > 90% is labeled as excellent, > 80% as good, > 70% as satisfactory, > 60% as sufficient, > 50% as critical, and < 50% as fail. The interquartile range (IQR) in this study ranged from 72% to 87%. The mean IQR was 84% in both the first and second visits in all children.

### Statistical analysis of MRI data

Statistical analyses of the MRI data were performed using SPM12. First, using GMV in the first and second visits, multiple regression analyses for each academic achievement score with age, sex, SES, and total intracranial volume (TIV) in each visit were used as covariates of no interest. For GMV changes, the first minus second image, a subtraction image of each participant was created using the Image Calculator implemented in SPM12. Furthermore, using these subtracted images, multiple regression analyses were performed for each academic achievement score using age, sex, and SES at the second visit, and average TIV of both visits as covariates of no interest. Due to the small sample size, the effects of sex differences on brain volumes were not examined, but were controlled for.

Statistical analyses were performed with a family-wise error (FWE) corrected *P* < 0.05 at the cluster level with uncorrected *P* < 0.001 at the voxel level. Whole brain voxels were explored without structural or functional region-of-interest (ROIs) because structural ROIs can include functionally distinct areas and functional ROIs can be challenging due to unclear boundaries of cortical regions, individual variability of anatomy and function, and nonlinear properties of ROIs (Liu 2011). The Montreal Neurological Institute (MNI) coordinates were used.

## Results

Demographics of participants in the first and second visits are shown in Table 1.

**Table 1:** Characteristics of children.

*N* (boys:girls)	77 (46:31)
Mean age 1st (range)	11.7 (9–16)
Mean age 2nd (range)	14.4 (12–18)
Mean IQ 1st (range)	107 (71–136)
Mean IQ 2nd (range)	110 (78–141)
Mean SES 1st (range)	8.0 (3.5–13.0)
Mean SES 2nd (range)	8.0 (3.5–13.0)

IQ: intelligence quotient, SES: socioeconomic status.

### Academic achievement scores

The distributions of vocabulary, reading, writing, and arithmetic scores and their means are presented in Fig. 1. The Shapiro–Wilk test failed to show non-normality in vocabulary (*W* = 0.99, *P* = 0.65), writing (*W* = 0.98, *P* = 0.46), and arithmetic (*W* = 0.97, *P* = 0.06) scores, but showed non-normality in reading scores (*W* = 0.90, *P* = 0.004).

There were no significant differences between boys and girls in vocabulary (*t*[75] = 0.44, *P* = 0.66), reading (*t*[75] = 1.10, *P* = 0.28), or arithmetic (*t*[75] = 0.90, *P* = 0.37) scores. Girls showed higher writing scores than boys (*t*[75] = 2.22, *P* = 0.03).

The correlations between the achievement scores and demographics are shown in Table 2. As the SES in the first visit was almost the same as that in second visit, SES in the first visit was used for the correlations. The correlations between achievement scores (shown in gray), and those between achievement scores and IQ were observed.

**Table 2:** Correlations between variables.

	1	2	3	4	5	6	7	8
1. Vocabulary								
2. Reading	0.74^*^							
3. Writing	0.34	0.48^*^						
4. Arithmetic	0.52^*^	0.65^*^	0.38^*^					
5. IQ 1st	0.64^*^	0.64^*^	0.44^*^	0.51^*^				
6. IQ 2nd	0.66^*^	0.68^*^	0.42^*^	0.61^*^	0.79^*^			
7. Age 1st	−0.03	−0.01	−0.04	−0.10	0.13	0.02		
8. Age 2nd	−0.02	−0.04	−0.04	−0.12	0.13	0.00	0.95^*^	
9. SES 1st	0.46^*^	0.44^*^	0.44^*^	0.35	0.35	0.28	−0.08	−0.11

IQ: intelligence quotient, SES: socioeconomic status.

^^*^^ *P* < 0.05 with Bonferroni correction.

### Multiple regression analyses for achievement scores and brain volume

#### Gray matter volumes in the first visit and achievement scores

No significant associations were observed between GMV in the first visit and achievement scores in the second visit.

#### Gray matter volumes in the second visit and achievement scores

**Fig. 2:**
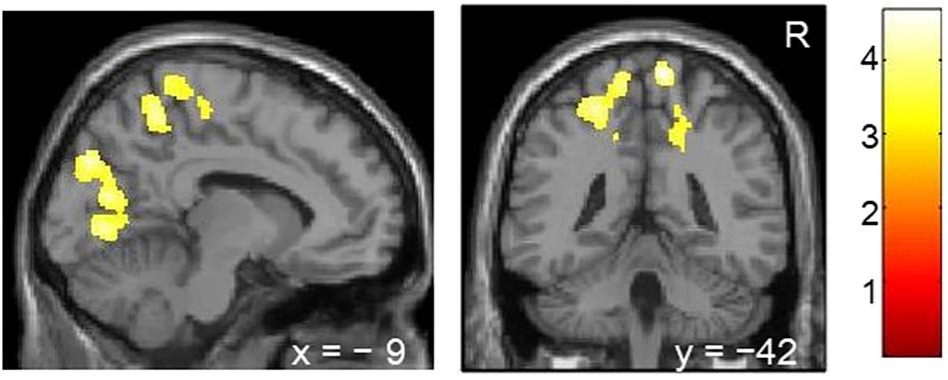
Associations between vocabulary score and cortical volume in the second visit. Higher vocabulary scores could be associated with smaller volumes in the precuneus and cuneus in the second visit. FWE-corrected *P* < 0.05 at cluster level with uncorrected *P* < 0.001 at voxel level. Age, sex, SES, total intracranial volume in the second visit are used as covariates of no interest. The color bar shows *z* value. *R* denotes right. *x* and *y* indicate MNI coordinates. SES: socioeconomic status.

Smaller cortical volumes in the precuneus and cuneus may be associated with higher vocabulary scores in the second visit (Fig. 2 and Table 3). No other associations were observed between GMV in the second visit and achievement scores.

**Table 3:** Current rGMV correlated with achievement scores.

				Corrected	Uncorrected			
			Peak	cluster	peak	Peak MNI coordinates		
	Brain areas	Voxels	*z* value	*P* value	*P* value	*x*	*y*	*z*
Vocabulary	Right precuneus	2,200	4.33	0.003	< 0.001	11	−42	75
	Left cuneus	2,175	4.14	0.003	< 0.001	−9	−83	35
	Left precuneus	2,179	3.97	0.003	< 0.001	−12	−50	54
Reading	−							
Writing	−							
Arithmetic	−							

Family-wise error corrected *P* < 0.05 at cluster level with uncorrected *P* < 0.001 at voxel level.

#### Changes in gray matter volumes and achievement scores

**Fig. 3:**
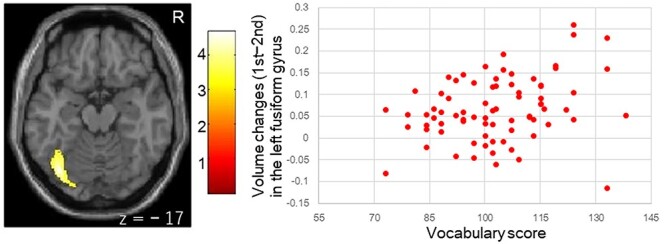
Greater cortical volume reduction over 2.6 years of development in children with high vocabulary scores. Children and adolescents with high vocabulary scores in the second visit exhibited greater rGMV reduction in the left fusiform gyrus. Relationships between vocabulary scores and the left fusiform volume changes (first minus second visit) are shown in the scatter plot. The color bar shows *z* value. Age, sex, SES at the second visit, and average total intracranial volume of both visits are used as covariates of no interest. *R* denotes right. *z* indicates MNI coordinates. SES: socioeconomic status.

Children with higher vocabulary scores showed a greater reduction in cortical volumes at 2.6 years in the left fusiform gyrus (Fig. 3 and Table 4).

**Table 4:** Changes in rGMV correlate with achievement scores.

				Peak MNI coordinates		
	Brain areas	Voxels	*z* value	*x*	*y*	*z*
Vocabulary	Left Fusiform Gyrus	1,158	4.48	−38	−72	−17
Reading	−					
Writing	−					
Arithmetic	Left Striatum	1,127	6.42	−14	11	0

Family-wise error corrected *P* < 0.05 at cluster level with uncorrected *P* < 0.001 at voxel level.

**Figure 4:**
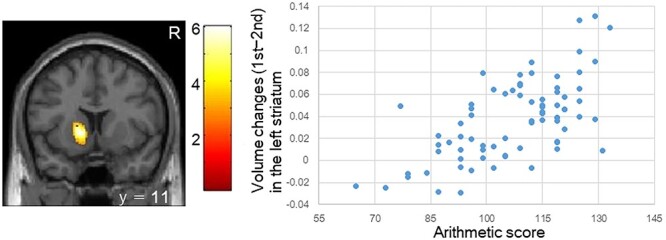
Children with higher arithmetic scores exhibited a greater reduction in the left striatal volume over 2.6 years of development than those with lower arithmetic scores. Relationships between arithmetic scores and striatal volume changes (first minus second visit) are shown in the scatter plot. Age, sex, SES in the first visit, and average total intracranial volume of both visits are used as covariates of no interest. The color bar shows *z* value. *R* denotes right. *y* indicates MNI coordinates. SES: socioeconomic status.

A greater reduction in left striatal rGMV was observed in children with high arithmetic scores than in those with low arithmetic scores (Fig. 4 and Table 4). No significant association with rGMV was observed for reading or writing. No significant increase in rGMV was observed for all of the 4 indices.

## Discussion

In this longitudinal, structural, MRI-based study, children and adolescents who scored higher on vocabulary and arithmetic tests exhibited a greater reduction in cortical volume. The current smaller brain volume could be associated with higher vocabulary scores, while brain volume in younger age groups showed no associations with all achievement scores. These results suggest that higher achievements in some academic skills may result from accelerated brain development, or vice versa, and that greater brain development may be associated with higher academic achievement.

Higher vocabulary scores were associated with smaller volumes of the precuneus and cuneus in the second visit. Visual object recognition, including imagery viewpoints in the precuneus, may enhance word–object binding and vocabulary (Cavanna and Trimble 2006; Slone et al. 2019; Tomasi and Volkow 2020).

Moreover, children and adolescents with higher vocabulary scores exhibited greater rGMV reductions in the left fusiform gyrus/ventral occipitotemporal cortex (vOTC). Less thickness of the left fusiform gyrus is associated with better recognition of irregular words that involves increased vocabulary in adults (Blackmon et al. 2010). Lesser volume in the left fusiform gyrus is also associated with better phonological awareness, and lesser fusiform surface area is related to better letter–word identification and phonological awareness in adults (Frye et al. 2010). In addition to visual word processing, letter-to-sound transformation in the fusiform network may be associated with the Japanese vocabulary with morphologically complex letters (Hashimoto et al. 2020). Greater development of the fusiform gyrus or vOTC may be related to greater knowledge of words in children.

Moderate correlations were found between the 4 academic achievement scores. These results suggest a common or overlapping mechanism in the vocabulary, reading, writing, and arithmetic achievements. However, the effects of higher scores in reading and writing on brain volume development could not be detected. As reading proficiency is observed in adults, reading scores may be associated with cortical volume later in adulthood (Torre and Eden 2019; Torre et al. 2020). Higher writing scores in girls may be a reason for the lack of a relationship between writing and brain volume with sex as a covariate of no interest; but, more samples are required to examine the gender gap (Reilly et al. 2019).

A reduced left striatal rGMV was observed in children with higher arithmetic scores than in those with lower arithmetic scores. The striatum is involved in incentive processes, motivation, and behavioral control (Delgado 2007; Somerville and Casey 2010). Procedural sequence learning in children is associated with striatal volume (Hedenius and Persson 2022). Striatal volumes peak at 10–12 years of age and reduce at adolescence (Narvacan et al. 2017; Duerden et al. 2020). Adolescents show differential involvements of the striatum in reward and motivation than adults (Silverman et al. 2015). In young adults, the striatum volume is associated with a belief that effort is rewarded (Hashimoto et al. 2015a). Therefore, positive attitudes and/or less aversion to arithmetic learning in children and adolescents may be associated with higher arithmetic achievement.

Changes in gray matter volume and cortical thickness concur in adults, but the surface area measurements show differential courses with thickness (Storsve et al. 2014). Age-related surface area expansion is localized to the cingulate gyrus, whereas age-related surface contractions are observed in many regions of the brain (Duerden et al. 2020). Age-related decreases in gray matter volume and thickness and comparably small decreases in surface area have been reported in children and young adults (Tamnes et al. 2017). Reading scores have been associated with cortical thickness, but not with surface area, in adolescents (Torre et al. 2020). However, surface area expansion has been shown to be positively associated with IQ (Schnack et al. 2015), and surface area measurement may provide further information about cognitive development.

This study has some limitations. The number of participants was insufficient to obtain a normal distribution in the reading score. The lack of association between brain volume and reading scores may have resulted from the non-normal distribution. The lack of data on academic achievement scores in the first visit limited the elucidation of the relationship between academic achievement and brain development (e.g. predicting brain development from previous achievement scores). In addition, the age range of the participants was large and may have obscured the effects of achievements on brain development. Further longitudinal studies in participants at young adulthood with narrow age ranges may provide a more comprehensive understanding of the academic achievement and cortical development in children.

In conclusions, the study suggested that higher vocabulary and arithmetic achievements may be derived from accelerated brain development, or that greater brain development may be associated with higher achievement. Higher vocabulary was associated with the development of the left fusiform gyrus, suggesting the involvement of word knowledge. The arithmetic scores were associated with the left striatum, highlighting the potential involvement of motivational factors. In summary, the study demonstrates that academic achievements correlate with brain development in children.

## Data Availability

All data generated and/or analyzed during this study are available from the corresponding author (TH) upon reasonable request.
